# Design and implementation of a microfluidic device capable of temporal growth factor delivery reveal filtering capabilities of the EGFR/ERK pathway

**DOI:** 10.1063/5.0059011

**Published:** 2021-11-02

**Authors:** Harris B. Krause, Hanna Bondarowicz, Alexis L. Karls, Megan N. McClean, Pamela K. Kreeger

**Affiliations:** 1 Department of Biomedical Engineering, University of Wisconsin-Madison, Madison, Wisconsin 53706, USA; 2 University of Wisconsin Carbone Cancer Center, University of Wisconsin School of Medicine and Public Health, Madison, Wisconsin 53705, USA; 3 Department of Cell and Regenerative Biology, University of Wisconsin School of Medicine and Public Health, Madison, Wisconsin 53706, USA; 4 Department of Obstetrics and Gynecology, University of Wisconsin School of Medicine and Public Health, Madison, Wisconsin 53705, USA

## Abstract

Utilizing microfluidics to mimic the dynamic temporal changes of growth factor and cytokine concentrations *in vivo* has greatly increased our understanding of how signal transduction pathways are structured to encode extracellular stimuli. To date, these devices have focused on delivering pulses of varying frequency, and there are limited cell culture models for delivering slowly increasing concentrations of stimuli that cells may experience *in vivo.* To examine this setting, we developed and validated a microfluidic device that can deliver increasing concentrations of growth factor over periods ranging from 6 to 24 h. Using this device and a fluorescent biosensor of extracellular-regulated kinase (ERK) activity, we delivered a slowly increasing concentration of epidermal growth factor (EGF) to human mammary epithelial cells and surprisingly observed minimal ERK activation, even at concentrations that stimulate robust activity in bolus delivery. The cells remained unresponsive to subsequent challenges with EGF, and immunocytochemistry suggested that the loss of an epidermal growth factor receptor was responsible. Cells were then challenged with faster rates of change of EGF, revealing an increased ERK activity as a function of rate of change. Specifically, both the fraction of cells that responded and the length of ERK activation time increased with the rate of change. This microfluidic device fills a gap in the current repertoire of *in vitro* microfluidic devices and demonstrates that slower, more physiological changes in growth factor presentation can reveal new regulatory mechanisms for how signal transduction pathways encode changes in the extracellular growth factor milieu.

## INTRODUCTION

The cellular microenvironment is dynamic with changes in the production, transport, and degradation of growth factors and cytokines, leading to complex presentation of these stimuli over time. These temporal patterns are sensed by cellular receptors and encoded to signaling hubs, such as extracellular-regulated kinase (ERK), a serine/threonine protein kinase that plays a major role in human development and disease by regulating fundamental cellular processes such as apoptosis,^1,2^ proliferation,^10^ and collective cell migration.^5^ A canonical activator of ERK is the epidermal growth factor (EGF)/epidermal growth factor receptor (EGFR) interaction, in which EGF binding to EGFR leads to EGFR homo- and hetero-dimerization, recruitment of Grb2 and son of sevenless (SOS), activation of RAS, and downstream phosphorylation of RAF, MEK, and ultimately ERK. Numerous negative and positive feedback mechanisms regulate the EGFR/ERK pathway, providing opportunities for the pathway to encode different presentations of EGF (e.g., amount, timing) into patterns of ERK activity.

Despite the dynamic nature of the *in vivo* microenvironment, the majority of studies of EGFR/ERK have utilized tissue culture plates with static wells. In this setting, a set concentration of EGF is applied to cells, ERK is quickly phosphorylated, and the amount of phosphorylated ERK shows a dose-dependency with respect to the initial EGF concentration.^6^ While often interpreted as a constant concentration of EGF throughout the experiment, ligand depletion in this paradigm will lead to a decrease in EGF concentration over time.^7^ To overcome this complication, cells have been plated as a small island in a well plate, such that ligand depletion relative to the initial EGF concentration was neglible.^8^ With this modification, it was observed that as EGF concentration increased, ERK pulsed on and off in individual cells with a greater frequency.

Recapitulation of the *in vivo* environment to understand EGFR/ERK requires delivery of well-defined increasing, decreasing, and pulsatile concentrations of EGF, which is difficult in tissue culture studies. Microfluidic approaches provide a promising approach to mimic the dynamic temporal presentation of growth factors as small volumes of fluid can be easily manipulated and exchanged. Devices have been developed to deliver pulses of growth factor to cells,^8–10^ and these studies have successfully identified the regulatory strategies employed by signal transduction pathways. For example, cells treated with a step change of EGF achieved a maximum ERK activity at similar times but returned to baseline faster as EGF concentration increased, suggesting a dose-dependent negative feedback mechanism.^9^

However, one aspect of how ERK encodes temporal stimulation that has not been well studied is how the EGFR/ERK pathway responds to more gradual increases in EGF concentration. Enhancing our understanding of how the ERK pathway encodes increasing concentrations of a growth factor is particularly important as growth factor concentrations increase over time in the developmental and wound^11^ microenvironments. Microfluidic systems can achieve time-varying concentrations by mixing fluid streams from different reservoirs, but often have difficulty achieving shallow gradients and require expensive or custom-built flow-control instrumentation.^10,12–14^ Using a gravity-driven microfluidic device, Mokashi *et al.*^12^ delivered an increasing concentration of tumor necrosis factor (TNF) to cells over timeframes of up to 8 h and found that the level of NF-κβ activation increased as the concentration of TNF increased. However, culture for extended periods of time (>5 h) was challenging in this system due to the formation of bubbles, and the system as designed only enabled one experimental condition at a time, limiting the ability to look at how increasing concentrations are encoded to ERK signals and decoded to slower processes, such as proliferation and differentiation.

Here, we develop and validate a microfluidic device that can deliver increasing concentrations of a stimuli over the course of hours to days, allows for up to four devices to be imaged simultaneously, incorporates a degassing protocol and a bubble trap to prevent bubbles, and utilizes readily accessible and inexpensive syringe pumps. These modifications enabled us to examine how a range of EGF ramps impacted ERK signaling.

## RESULTS

### Design and validation of microfluidic device

**FIG. 1.**
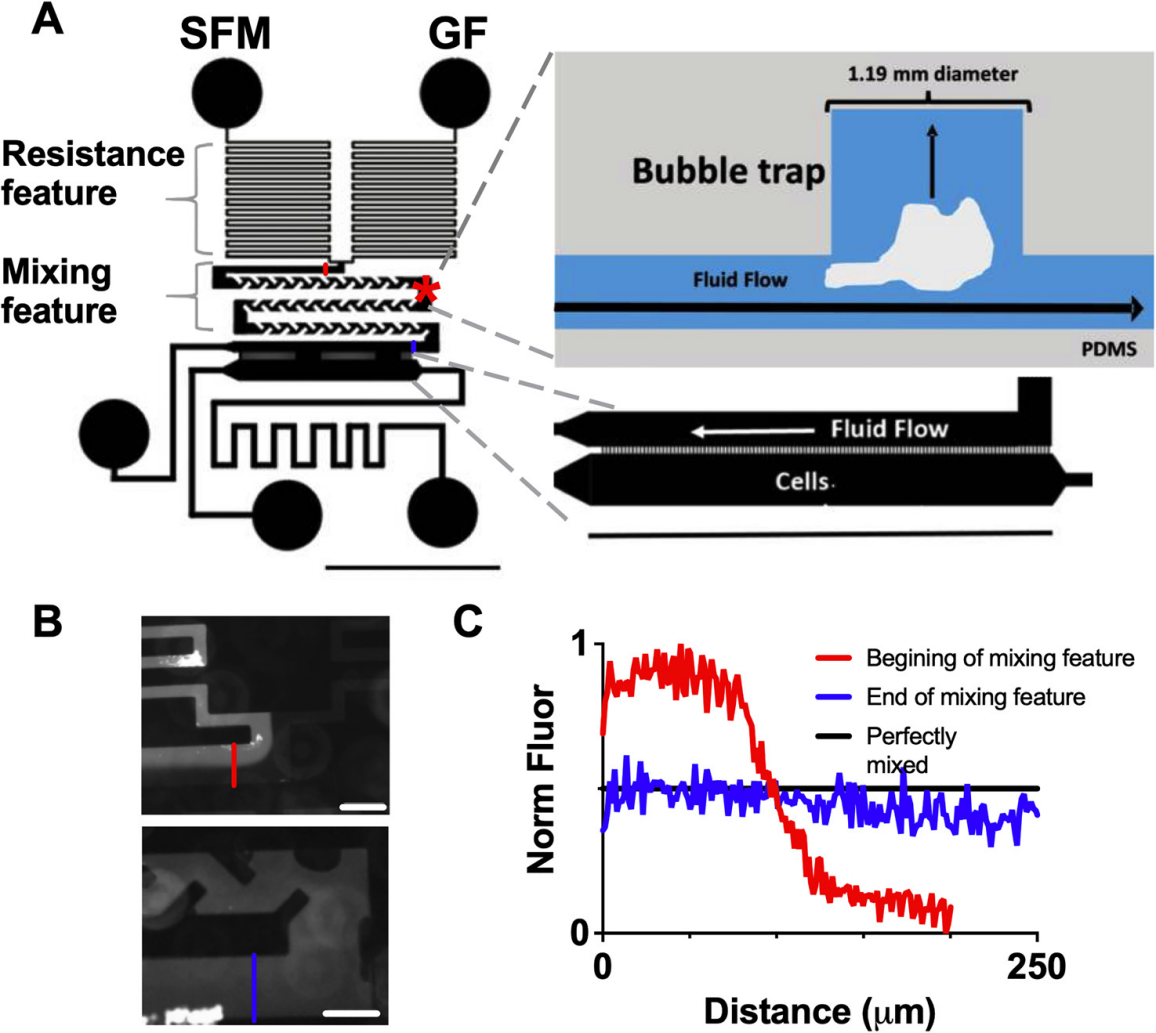
(a) Schematic of microfluidic device with close-up of the cell chamber. The asterisk denotes the bubble trap location. Scale bar = 5 mm. (b) Images of fluorescently tagged dextran at the beginning (top panel) and end (bottom panel) of mixing feature. Scale bar = 200 *μ*m. (c) Normalized fluorescent intensity at the beginning and end of mixing feature [distance is the distance across the cell chamber at locations marked in (c) with the same color; locations for these imaging spots also noted in (a) with same color scheme].

The device consists of two inputs [serum free media (SFM) and SFM + growth factor (GF)] driven by programmable syringe pumps that flow through a resistance feature to minimize flow variation [Figs. 1(a) and S1]. Next, the two streams pass through a mixing element where they mix through both diffusion and convection.^27^ A bubble trap was included in the mixing region to reduce the occurrence of bubbles that can shear cells [Fig. 1(a)]. The mixed streams then flow past a series of diffusion ports (50 × 15 *μ*m^2^ spaced at 30 *μ*m intervals) connected to a cell chamber where cells were seeded (600 *μ*m × 5 mm).^3^ The ports allow diffusion while minimizing fluid flow over the cells in order to lessen the effects of shear stress, including ERK activation.^8,28^ The syringe pumps were programmed to change the relative flow rate of the SFM and GF streams (while keeping the overall flow rate constant), allowing increasing concentrations of growth factor to be delivered. We validated mixing in the device using 10 kDa fluorescently tagged dextran, which has a molecular weight comparable to many cytokines and growth factors (including EGF, 6.4 kDa). Each stream was set to a flow rate of 0.18 *μ*l/min, with 100 nM of labeled dextran in the GF stream and SFM in the other stream. Using a fluorescence microscope, two distinct streams were observed at the beginning of the mixing feature, but a near-uniform stream was present at the end [Figs. 1(b) and 1(c)].

**FIG. 2.**
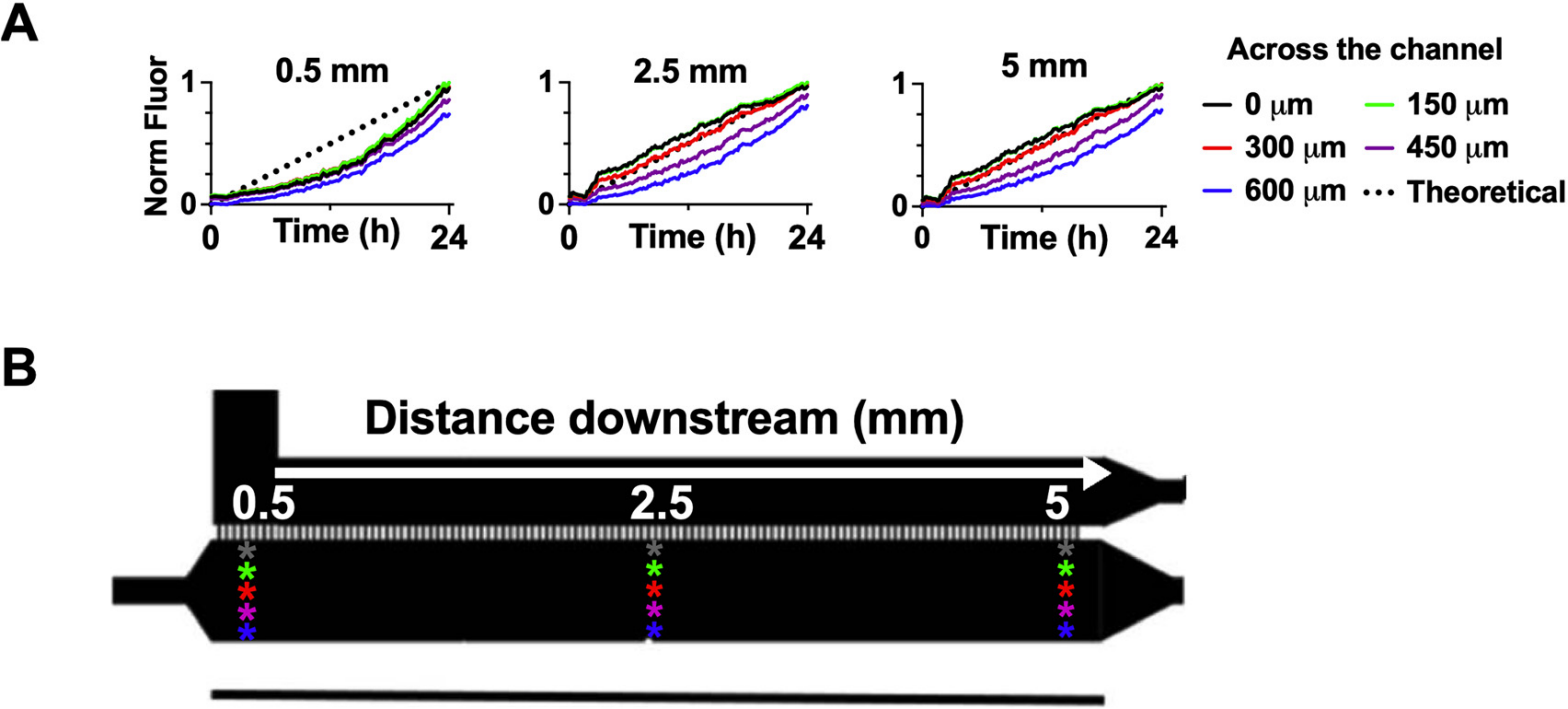
(a) Quantification of increasing concentration based on the fluorescent dextran signal at three distances along the cell chamber (0.5, 2.5, and 5 mm from beginning) and across the cell chamber (0, 150, 300, 450, and 600 *μ*m from the diffusion ports at that location). (b) Schematic of analyzed locations, white arrow indicates direction of flow.

To determine how quickly the cell chamber would reach equilibrium with the fluid stream, COMSOL simulations were performed for a cell chamber filled initially with SFM as media with either 1 or 100 ng/ml EGF were flowed through the device at a flow rate of 0.36 *μ*l/min. For both concentrations, the cell chamber was fully equilibrated after three minutes [Figs. S2(A) and S2(B)]. The simulation demonstrated that due to the diffusion ports, there was minimal fluid velocity in the cell chamber at steady state [fully developed flow, Fig. S2(C)]. We experimentally validated the ability of the device to ramp up rapidly and deliver steady concentrations of EGF [Figs. S2(D)–S2(H)]. We next examined if the device could deliver an increasing temporal gradient. Linear slopes were selected and fluorescently tagged dextran was utilized to examine if the desired gradients were delivered. Multiple positions were measured both across and along the cell chamber to examine the potential spatial variation. To identify the time frames where a smooth change in concentration could be implemented, linear ramps over 3–24 h were tested. For ramps over six to 24 h, minimal differences in fluorescent intensity were observed with increasing distance from the diffusion ports at three points along the cell chamber [Figs. 2 and S3(A)], demonstrating that cells seeded at different positions within the chamber will experience similar concentrations of growth factor. The largest variation from the desired linear ramp was observed at the point furthest from the diffusion ports at the beginning of the cell chamber, suggesting that this corner of the cell chamber takes additional time to equilibrate. The device could deliver decreasing [Figs. S3(B)–S3(F)] in addition to increasing concentrations with minimal fluctuations in signal relative to the targeted linear slope. Finally, to demonstrate that we can deliver increasing, decreasing, and steady concentrations within one device, a combination of different patterns was administered that saw good concurrence with the theoretical delivery pattern [Fig. S3(G)]. While adsorption of small, hydrophobic compounds is a known challenge with polydimethylsiloxane (PDMS) devices,^29^ the close agreement between the planned and observed pattern demonstrated that there was minimal adsorption of large, hydrophilic molecules. While prior devices have been developed to deliver ramps on the length scale of minutes to a few hours,^10,12^ our device can achieve smooth ramps over significantly longer time frames (up to 24 h) without compromising the cell viability [Fig. S2(I)].

**FIG. 3.**
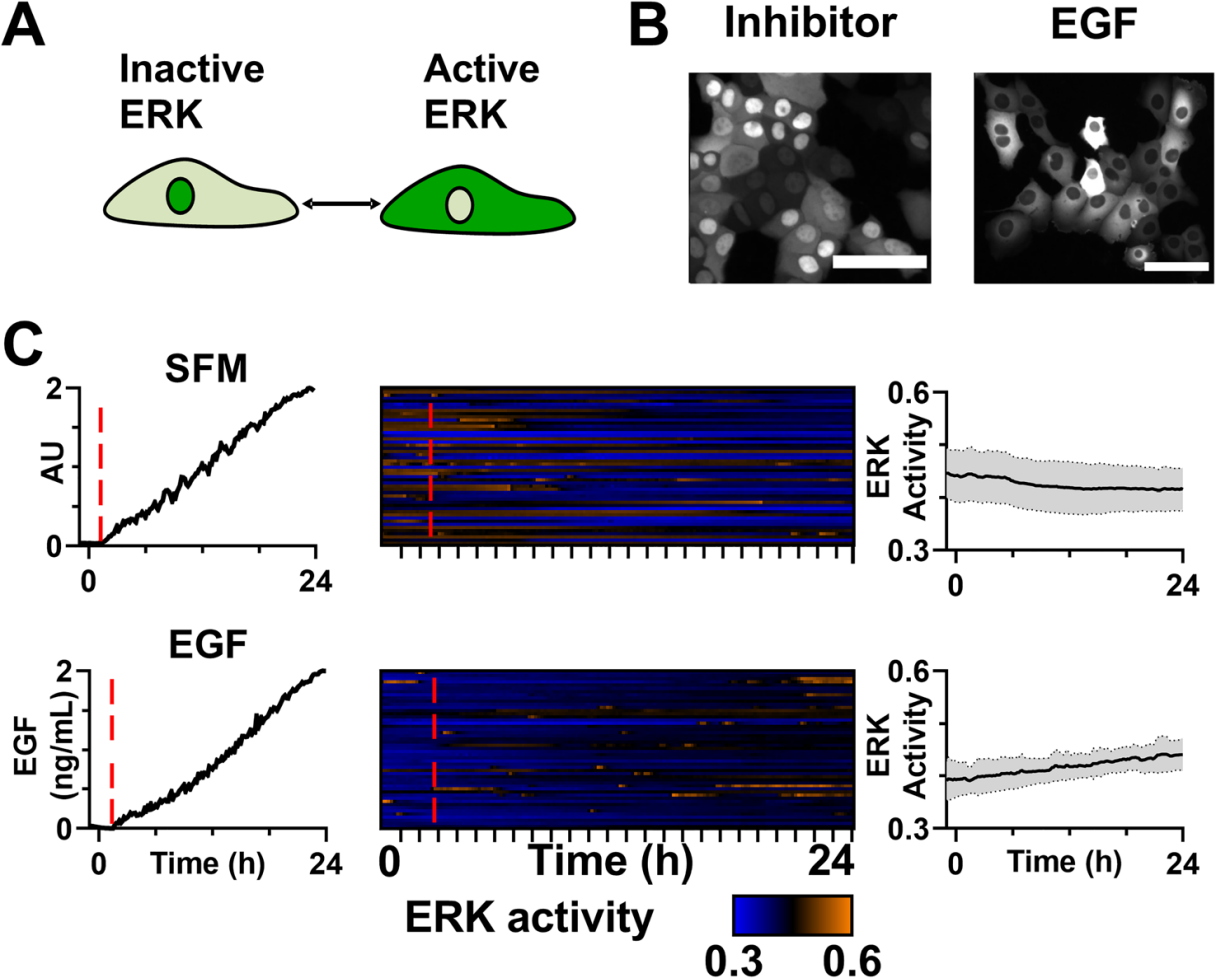
(a) Schematic of ERKKTR reporter. (b) Images of ERKKTR when cells are dosed with ERK inhibitor (FR180204) or 2 ng/ml EGF. Scale bar = 100 *μ*m. (c) Linear dosing confirmed by fluorescently tagged dextran (left), heatmap of single cell ERK activity for cells (middle), and average ERK activity (right) for linear ramps of SFM→SFM and SFM→2 ng/ml EGF over 24 h. Due to device-to-device variation, the time that the EGF ramp begins is denoted by a red dashed line. The average activity is shown as a black line with the gray shading demonstrating the standard deviation. n = 50 cells. Two independent repeats were conducted and showed the same trend.

### ERK fails to activate in response to slow increases in EGF

We next utilized this device to examine how cells encode slow increases in EGF into ERK signaling in HMECs, a benign human mammary epithelial cell line. To measure ERK activity at a single-cell level, we used the ERKKTR fluorescent biosensor.^4^ When ERK is inactive, ERKKTR localizes to the nucleus and when ERK is active, ERKKTR localizes to the cytoplasm [Fig. 3(a)]; H2B-RFP was utilized to identify the nuclear region in images. To confirm the ERKKTR biosensor was working as expected, HMECs were dosed with either 60 *μ*M ERK-inhibitor FR180204 or a 2 ng/ml bolus dose of EGF in a standard culture well. ERKKTR localized to the nucleus when ERK was inhibited and to the cytoplasm when ERK was activated [Fig. 3(b)]. A ratio between the fluorescent intensity in the cytoplasm vs the combined signal in the cytoplasm and nuclear regions [C/(C + N)] provides a quantitative measure of ERK activity, with a higher ratio indicating more ERK activity. We next examined the ERK activity in the device by exposing the cells to step changes from SFM to the GF channel containing either SFM, 1 ng/ml EGF, or 10 ng/ml EGF (Fig. S4). We found that abrupt steps could generate enough shear stress to transiently activate the ERK pathway, even when the second channel contained only SFM. This effect was not observed when the fluid streams changed from 0 to 0.36 *μ*l/min over 24 h; however, to monitor for the potential effects we performed SFM to SFM control ramps in all experimental conditions.

**FIG. 4.**
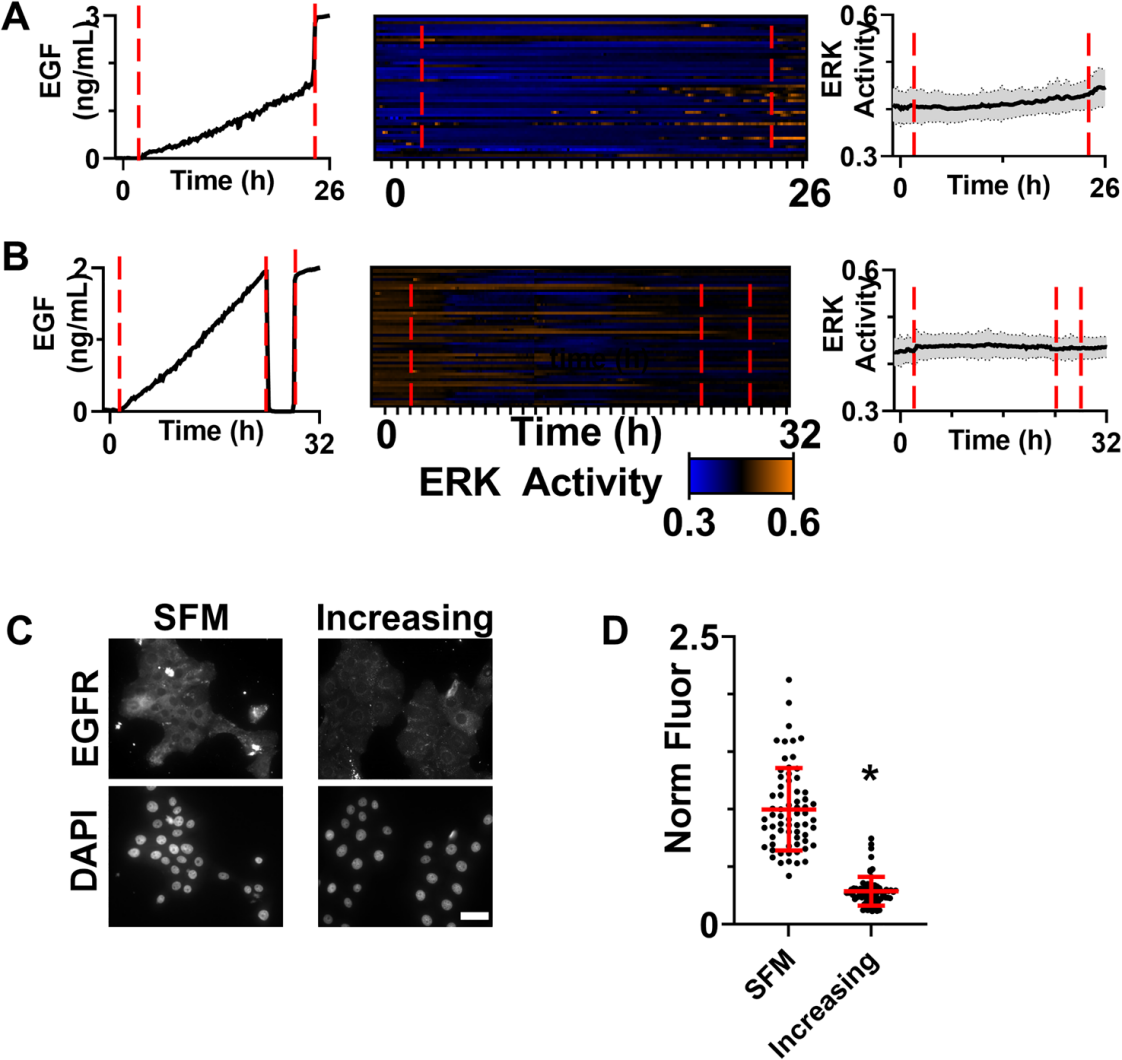
(a) ERK activity patterns in response to a slow ramp of EGF from 0 to 2 ng/ml over 24 h followed by a step increase to 3 ng/ml. Shown are dosing confirmed by fluorescently tagged dextran (left), heatmap of single-cell ERK activity for cells (middle), and average ERK activity (right). Due to the device-to-device variation, the time that increasing concentration begins is denoted by a red dashed line. The subsequent transition to 3 ng/ml is also denoted with a red dashed line. The average activity is shown as a black line with the gray shading demonstrating the standard deviation. n = 50 cells. (b) ERK activity patterns in response to a slow ramp of EGF from 0 to 2 ng/ml, a 4 h period of SFM, and a return to 2 ng/ml EGF. Shown are dosing confirmed by fluorescently tagged dextran (left), heatmap of single-cell ERK activity for cells (middle), and average ERK activity (right). Due to the device-to-device variation, the time that the increasing concentration begins is denoted by a red dashed line. The subsequent transitions to 0 ng/ml and back to 2 ng/ml are also denoted with red dashed lines. The average activity is shown as a black line with the gray shading demonstrating the standard deviation. n = 50 cells. (c) HMECs stained for EGFR after exposure to SFM or 0 to 2 ng/ml EGF over a 24 h period. Scale bar = 50 *μ*m. (d) Quantification of EGFR staining intensity for individual cells. Each cell indicated by a dot bars represent the mean ± SD. Asterisk indicates p < 0.05. n = 66 cells (SFM), 71 cells (increasing EGF). For (a), (b), and (c) two independent repeats were conducted and showed the same trend.

Next, HMECs in the microfluidic device were exposed to a linear increase from 0 to 2 ng/ml EGF over 24 h. A SFM control was also conducted where cells were exposed to an increasing concentration of SFM containing fluorescently tagged dextran. The increasing concentration was successfully delivered in both conditions as indicated by the fluorescent signal from the dextran [Fig. 3(c), left]. Cells exposed to the SFM control demonstrated only a small drift in their ERK activity and did not show any shear-related effects (median change in ERK activity for SFM = −0.01, p < 0.001, paired t-test of individual cells at t = 0 vs 24 h). We observed only minimal activation of ERK in the 0 to 2 ng/ml increasing condition (median change in ERK activity for EGF = 0.04, p < 0.001, paired t-test of individual cells at t = 0 vs 24 h). This was surprising, as 2 ng/ml activated a strong ERK signal when delivered instantaneously [Fig. 3(b)].

### Increasing concentrations of EGF result in loss of sensitivity to subsequent EGF stimulation

Two possible explanations for the lack of ERK activity in response to slowly increasing EGF are negative feedback within the ERK pathway^8,30^ or internalization/degradation of EGFR.^31,32^ In many negative feedback loops, activation of the pathways leads to an increase in the level and/or activity of a negative regulator. Such inhibition can be overcome with a dose of the stimulant that exceeds the inhibition threshold or that remains present after the inhibition has been released through mechanisms, such as inhibitor degradation. To attempt to exceed the inhibition threshold, we exposed HMECs to an increasing concentration of EGF (0 to 2 ng/ml) followed by a spike to 3 ng/ml [Figs. 4(a) and S5(A) for SFM control]. HMECs have been shown to express approximately 1 × 10^5^ EGFR per cell in HMECs;^19^ therefore, for our cell density of 1000 cells/device, the expected number of EGFR copies per device is approximately 10^8^ receptors. At a flow rate of 0.36 *μ*l/min and 3 ng/ml EGF, the device delivers sufficient EGF to saturate the receptor within 1 min. However, ERK activation was not observed at a population level in response to this spike; instead, only a few cells that had active ERK during the ramp period continued to have ERK activation, suggesting that the inhibition was strong enough to filter out a 50% increase in stimulation. As shown in Fig. S5(A), we did not observe substantial ERK activation when this was done with a SFM control. Protein half-lives in proliferating mammalian cells have been shown to be generally less than 2 h;^33^ therefore, cells were exposed to the slowly increasing concentration of EGF followed by a four-hour holiday and then a sudden return to 2 ng/ml EGF [Fig. 4(b) and S5(B) for SFM control]. Again, few cells showed a response to EGF after the holiday, and the population average activity was not significantly different than the level prior to the holiday (p = 0.42) or prior to the second stimulation (p = 0.35), suggesting that the timescale of inhibition is longer than 4 h.

**FIG. 5.**
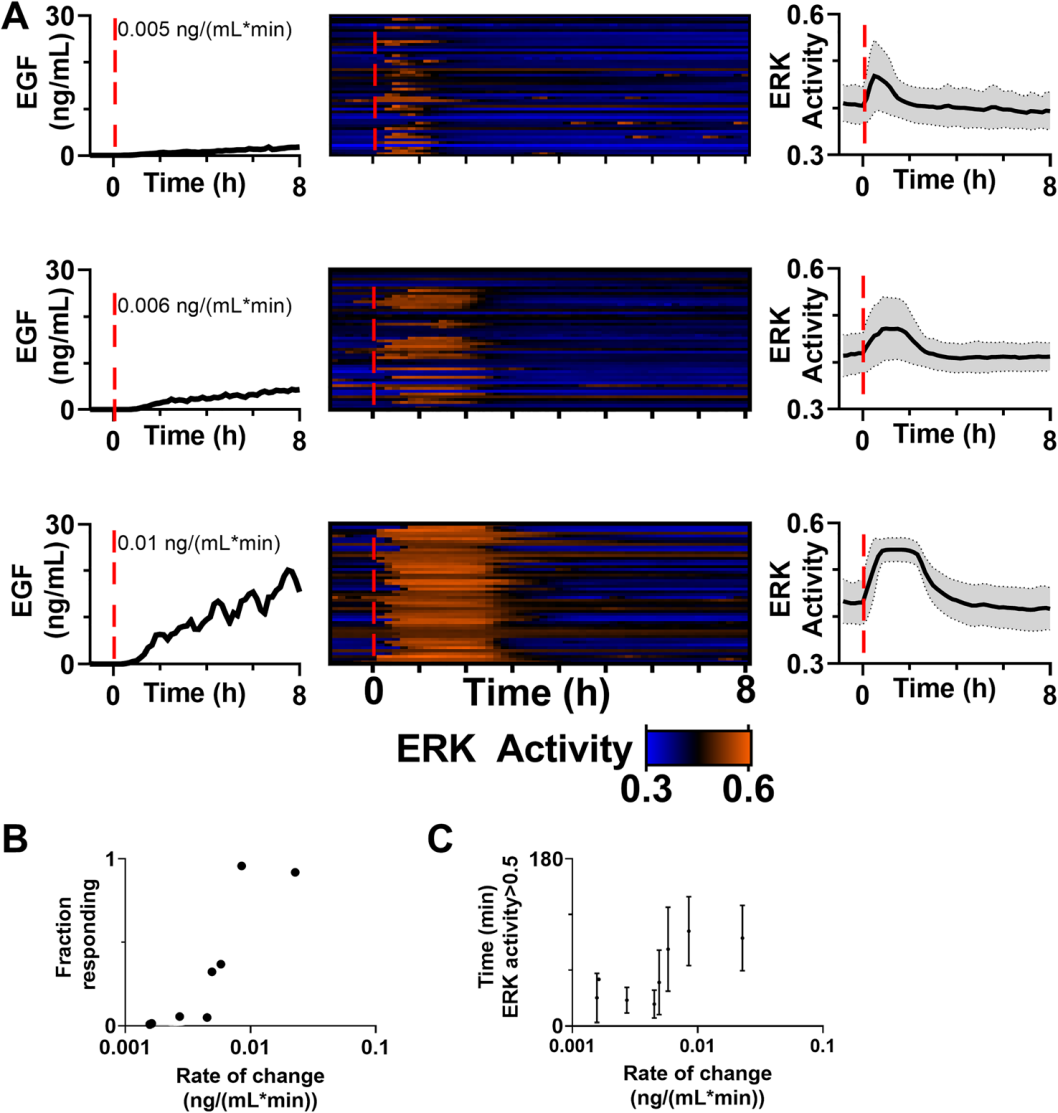
(a) ERK activity patterns in response to a varying the rate of change of EGF. Shown are dosing confirmed by fluorescently tagged dextran (left), heatmap of single-cell ERK activity for cells (middle), and average ERK activity (right). All time scales were adjusted so t = 0 when dextran (EGF) was first detected (also denoted by a red dotted line). (b) and (c) ERK activity patterns for cells over a range of rates of change, with ERK activity >0.5 classified as an active cell. Both the fraction of cells with active ERK (b) and the time in this active state (c) show a switch-like pattern with respect to the initial rate of change (b) k_d_ = 0.0060, hill slope = 7.12, $R^{2}=0.97$ (c) k_d_ = 0.0050, hill slope = 11.27, $R^{2}=0.23$. Dots represent the fraction (b) or average time (c) of all the cells analyzed for a particular rate of change. In (c), bars indicate the SD. (b) and (c) also include the data from Fig. 3. n > 50 cells/condition.

To identify the source of inhibition, we started at the receptor level of the pathway. Cells were exposed to an increasing concentration of EGF for 24 h, fixed, and then stained for EGFR using a protocol optimized for quantitative immunostaining for cells cultured in this device [Fig. 4(c)]. Single-cell quantification of EGFR expression [Figs. 4(d) and S6] demonstrated significantly lower levels of EGFR expression in cells exposed to an increasing concentration of EGF as compared to the SFM control, suggesting that loss of the EGFR in response to increasing EGF resulted in pathway insensitivity. As a test of this potential mechanism, we repeated our experimental paradigm using HaCaT keratinocytes, which express approximately 8.6 × 10^5^ EGFR per cell^34^ compared to 1 × 10^5^ EGFR per cell in HMECs.^19^ At this much higher level of EGFR, we would not expect our low concentrations to induce sufficient loss of receptor to dampen the signal. As predicted, we see a small, but consistent, activation of ERK in response to the 0 to 2 ng/ml ramp in HaCaTs [supplementary material Figs. S7(A) and S7(B)].

### The rate of change of EGF is encoded by both the fraction of responding cells and length of response

Given that ERK is activated in response to step changes of EGF [Fig. 3(b)], but only minimally in response to gradual ramps (Fig. 4), we utilized the ability of the device to vary the rate of change and monitored ERK activation in response [Fig. 5(a)]. To keep the change in flow rate the same (transitioning from 0 to 0.36 *μ*l/min over 24 h), we varied the concentration in the second stream from 0.5 to 100 ng/ml. In contrast to the slowest ramp of 0.0016 ng/(ml min) (Fig. 4), we observed robust ERK activation at early times with faster ramps. Interestingly, it appeared that as the temporal gradient of EGF increased, the fraction of cells activated and the length of ERK activation increased [Fig. 5(a)]. Using the C/(C + N) threshold of 0.5 determined from our dynamic range analysis, we plotted the rate of change of EGF against the fraction of cells responding, defined as the percentage of cells that reach the C/(C + N) threshold for longer than 20 min during the first three hours after EGF begins to be delivered increasing concentration [Fig. 5(b)], and the length of time that ERK remained active in cells that responded [Fig. 5(c)]. Both the fraction of cells responding and the length of ERK activation increased as the rate of change increased. Intriguingly, HMECs demonstrated switch-like behavior as the rate increased above 0.005 ng/(ml min) (EC50 determined by Hill equation). HaCaTs that express eightfold more EGFR (HaCaTs) were exposed to increasing rates of change of EGF (Fig. S7). As HaCaTs showed ERK activation at all ramp rates, we did not observe switch-like behavior. However, like the HMECs, both the fraction of cells responding and length of ERK activation increased as the rate of change increased. Finally, to determine if the differences in ERK activation dynamics impact downstream targets, HMECs exposed to a slow and fast ramp were stained for FRA-1, a transcription factor that has been shown to be differentially expressed in response to different patterns of ERK activation.^35^ We observed a significantly increased expression of Fos-related antigen 1 (FRA-1) in cells that were exposed to a faster ramp and, therefore, had more ERK activity (Fig. S8).

## DISCUSSION

Using a device that we developed and optimized to deliver increasing concentrations of EGF over time, we determined that at very slow rates of change, the ERK network in HMEC cells is largely unresponsive. Conversely, as the rate is increased, a larger fraction of cells responded and the time with active ERK increased. These findings add to the complex variety of encoding mechanisms that have been observed for the growth factor/ERK pathway.

We first validated that our device delivers increasing concentrations of growth factor over time frames as long as 24 h. Prior attempts to mimic a regimen of gradual increases in growth factor concentration include a gravity-driven device that can deliver increasing concentrations over 5–8 h^12^ and devices that approximated increasing concentrations as a series of small step changes.^10^ An advantage of this device is that it utilizes syringe pump driven flow, which can be a more affordable setup compared to pressure pump systems. Our model simulations and experimental analysis demonstrated that the profile of growth factor in the device was uniform. While for us this was an advantage to maximize the number of cells that could be analyzed, further modification to add a parallel channel on the other side of the cell chamber might enable examination of spatial variations.^8,10^ While not tested in this work, we expect that the device could deliver combinations of growth factors that are all increasing at the same rate. While the time frame of 24 h is short relative to developmental processes, it is a similar timescale as the lifespan of an individual cell. Further device optimization to deliver longer temporal patterns would enable study of cellular “memory” of the growth factor temporal environment over multiple generations.

After validating the device, we examined ERK response in HMECs exposed to a slow increasing concentration of EGF (0 to 2 ng/ml over 24 h). Interestingly, we did not see substantial ERK activation in response to the slow ramp, even once the device had reached 2 ng/ml. Past work has found that even lower concentrations of EGF (1 ng/ml) delivered as a bolus dose activated HMECs.^36^ We hypothesized that this insensitivity resulted from negative feedback. To test the strength of this feedback, we increased the concentration of EGF by 50% at the end of the time course but were unable to activate ERK. Next, to test the duration of this feedback, cells were given a 4-h holiday prior to challenging with 2 ng/ml EGF. As this did not alter ERK signaling, we hypothesized that the feedback was not the presence of a negative inhibitor (which might degrade on that timescale), but rather the loss of a positive regulator. Immunostaining of cells after exposure to 24 h of gradually increasing concentrations of EGF supported this hypothesis, as cells exposed to SFM had significantly higher expression of EGFR as compared to cells exposed to increasing EGF. One possible explanation for the loss of EGFR in response to EGF is that the receptor was internalized and degraded. EGFR trafficking is an established mechanism to respond to receptor-level activation and is thought to act as a mechanism of signal attenuation.^37^ However, our results suggest it may also act as noise filter, filtering out small and slow changes in growth factor such that EGFR is internalized before the EGF reaches a high enough concentration in the local microenvironment to trigger ERK activation. Interestingly, this filtering function was not seen in the tested ramps in HaCaT cells, which express substantially more EGFR.

While cells were able to filter out a slow ramp from 0 to 2 ng/ml over 24 h, ERK activation is seen in response to step increases, such as bolus dosing in plates in our work and many others.^14^ This suggests that there must be a rate of increasing concentration that is high enough to escape the potential for filtering through loss of EGFR. To test this, we increased the temporal gradients of EGF in both HMEC and HaCaT cells and found that both the fraction of cells responding, and the length of response increased. While prior studies have examined ERK signaling in PC12 cells in response to increasing concentrations of EGF delivered into a well plate by syringe pumps, fractional responses with respect to increasing rate of change were not observed, likely due to the use of rates above our switch point [0.008 to 0.08 ng/(ml min)].^14^ To achieve our different ramps, we increased the concentration of the growth factor in the second stream; it is, therefore, possible that our observation was dependent on concentration rather than the rate of change. However, when the rate of change was greater than 0.005 ng/(ml h) we observed robust activation by 1 h, which corresponds to a concentration of approximately 0.7 ng/ml. In contrast, when the rate of change was less than 0.005 ng/(ml h) we did not see clear activation by 8 h, where the concentration of EGF was also around 0.7 ng/ml. Therefore, while it remains likely that the concentration is an important factor in pathway activation, all conditions tested here experienced similar instantaneous concentrations at some point, suggesting that the rate is also an important feature. A recent report has demonstrated that the high osmolarity glycerol (HOG) MAPK in yeast is also sensitive to the rate at which osmotic stress is applied.^38^ It is likely that slow increases in osmostress do not immediately threaten cellular fitness and can be compensated for by existing cellular mechanisms to restore turgor pressure that do not require HOG pathway activation; therefore, a rate threshold may serve to conserve cellular resources for more significant perturbations. Intriguingly, the HOG pathway ramp sensitivity comes from a negative feedback mechanism whereas one of the mechanisms at play here involves pathway desensitization through receptor loss.

The observation of extended ERK activation in response to EGF is in contrast to much of the literature where EGF results in short peaks (<1 h) and other growth factors, such as nerve growth factor (NGF) lead to longer activity (>1h).^8,14^ Prior studies have demonstrated that the expression of some ERK-target genes, such as *FOSL1* (the gene for FRA-1), act as linear integrators of ERK activity;^35^ similarly, we observed increased FRA-1 in cells that were exposed to faster ramps and therefore longer periods or ERK activity. Of course, the precise interpretation of ERK signaling patterns may depend on the target gene.^39^ To our knowledge, no study has addressed how the length of EGF-induced ERK activation decodes to cellular phenotypic decisions. However, cells can continuously sense kinase signaling throughout the cell cycle and a recent study suggested that cells integrate this activity into decisions to proliferate.^40^ In many treatment regimens, asynchronous pulses of ERK activity are observed.^3,41–43^ To understand the effect of this encoding, optogenetics has been used to mimic pulsatile ERK, showing that intermittent ERK activation leads to a higher rate of cell proliferation as compared to continuous activation.^41^ Additionally, in an elegant system that used optogenetic control of ERK in addition to fluorescent reporters of transcription, multiple pulses of ERK were shown to induce immediate early genes more efficiently than sustained activity, suggesting that ERK activation length will alter gene transcription patterns,^44^ as we observed for FRA-1.

In conclusion, our results demonstrate that the EGFR/ERK pathway responds to the rate at which EGF concentration increases. At the slowest rates, the pathway can filter out the stimuli based on the loss of the EGFR. As the rate increases, both the number of cells showing ERK activation and the length of ERK activity increase. Intriguingly, we observed a switch-like sensitivity to EGF rate in HMECs. Cells experience a variety of temporal patterns of growth factor and cytokine *in vivo*; therefore, studies of signal transduction pathway encoding should include the full array of these presentations to better decipher the potential responses.

## METHODS

All materials were purchased from Thermo Fisher Scientific (Waltham, MA) unless noted otherwise.

### Device fabrication and preparation

Soft lithography was used to fabricate a three-layer mold. First, silicon wafers (Pure Wafer, San Jose, CA) were spin coated with 20 *μ*m SU-8 25 (Microchem, Newton, MA), prebaked for 3 min at 65 °C followed by a soft bake for 7 min at 65 °C. Next, the wafer was exposed for 15 s to UV at 150 mJ/cm^2^ using a photomask. The photomask was aligned by hand using alignment features. The wafer was baked post-exposure for 1 min at 65 °C followed by 3 min at 95 °C. Once baked, wafers were developed by submerging in SU-8 developer (propylene glycol monomethyl ether acetate, PGMEA, 537542, Sigma, St. Louis, MO) with gentle agitation. This process was repeated for the second and third layers of the mold with SU-8 100 (80 and 400 *μ*m). The second layer was prebaked for 10 min at 65 °C followed by a soft bake for 30 min at 65 °C, exposure to UV at 240 mJ/cm^2^ for 15 s, baked post-exposure for 1 min at 65 °C and 10 min at 95 °C, and developed in PGMEA. The third layer was prebaked for 30 min at 65 °C followed by a soft bake for 90 min at 65 °C, exposure to UV at 370 mJ/cm^2^ for 15 s, baked post-exposure for 1 min at 65 °C and 20 min at 95 °C, and developed in PGMEA. 10 ml of uncured polydimethylsiloxane (PDMS, mixed in a 10:1 ratio of monomer to curing agent, Krayden Dow Sylgard 184 elastomer kit) was poured into the mold and polymerized for 2 h at 65 °C. Devices were soaked in isopropanol for 20 min to remove uncured monomer and clean the surface and then baked in an oven at 65 °C for 20 min. PDMS blocks of 4 mm height were dipped in uncured PDMS, placed on top of the media inlets and site of the bubble trap^15^ [ESI Fig. S1(A)], and then the devices were baked for 2 h at 65 °C. Inlets and the bubble trap were then punched out with an 18-gauge blunt needle, and the cell seeding and outlet ports were punched out with a 14-gauge blunt needle (SAI Infusion technologies, Lake Villa, IL). A 20 *μ*l pipette tip (VWR, Radnor, PA) was inserted from the bottom of the device halfway through the bubble trap hole made by the blunt needle. Uncured PDMS was placed on top of the bubble trap and allowed to fill the part of the hole not filled by the pipette tip. Devices were baked at 65 °C for 2 h. The pipette tips were removed, and devices were cleaned by soaking in isopropanol for 20 min and baked for an additional 20 min at 65 °C. Devices were plasma bonded to a glass coverslip (75 × 50 mm^2^, 1 mm thickness, Corning, Corning, NY) in two rows of four devices each [ESI, Fig. S1(B)]. Devices bonded to the coverslips were sterilized for 20 min under UV light and baked at 65 °C for 2 h.

### Comsol simulation

Comsol Multiphysics 4.2^®^ was used to predict shear stress and growth factor concentrations in the cell chamber after 3 min of flow. A 2D version of the microfluidics device was used to simulate fluid flow and growth factor diffusion. Cell culture media was approximated as water (*ρ* = 1000 kg/m^3^, *μ* = 1 cP) and laminar flow conditions were assumed. The molecular weight of the diffusive component was set at 6.4 kDa (the MW of EGF^16^) with the diffusion coefficient set to 16.6 × 10^−7^ cm^2^/s.^16^ At the beginning of the simulation, there was no flow and no solute present in the device. Fluid entered the inlet at a velocity of 0.001 m/s and a concentration of 15.6 or 0.156 nM (equivalent to 100 and 1 ng/ml EGF). The shear stress and concentration of solute in the cell chamber were recorded after 3 min of flow.

### Production of virus for transduction

Lenti-virus was produced for pLentiCMV Puro DEST ERKKTRClover,^17^ a gift from Markus Covert (Addgene plasmid # 59150) and pHIV-H2BmRFP,^18^ a gift from Bryan Welm & Zena Werb (Addgene plasmid # 18982). Human embryonic kidney 293 T cells, cultured in DMEM/F12 supplemented with 1% Pen/Strep and 10% fetal bovine serum (FBS), were transfected using the Lenti-vpak Lentiviral Packing Kit (Origene, Rockville, MD) with the H2BmRFP and ERKKTRmClover vectors. After 24 h, the viral supernatant was collected and spun down for 10 min at 3000 RPM.

### Cell culture

Human mammary epithelial cells (HMECs 184a1, a gift from H. Steven Wiley, Pacific Northwest National Laboratory) were selected for use in this study. A key feature of this cell line is that while they do produce some EGF-family ligands, they do not produce autocrine EGF^19^ and express an order of magnitude more EGFR compared to HER2 and HER3.^20^ These features result in a simpler cellular system to study EGF signaling than many cell lines. HMECs were maintained at 37 °C, 5% CO_2_ in full-serum media, which was made up of a 1:1 (v/v) ratio of MEM Alpha and Ham's F-12 media supplemented with 2-[4-(2-hydroxyethyl)piperazin-1-yl]ethanesulfonic acid (HEPES) buffer (MilliporeSigma, St. Louis, MO) to a final concentration of 40 mM with 1% Pen/Step, 1 *μ*g/ml cholera toxin (MilliporeSigma), 10 *μ*g/ml insulin (MilliporeSigma), 1% fetal bovine serum (FBS), 3 *μ*g/ml bovine pituitary extract, 1 *μ*g/ml epidermal growth factor (EGF), 1 *μ*g/ml hydrocortisone (MilliporeSigma), 0.1 mM ethanolamine/phosphoethanolamine (MilliporeSigma), 10 *μ*g/ml Apo-transferrin (MilliporeSigma), 10 *μ*g/ml sodium selenite (MilliporeSigma), 1 *μ*M triiodothyronine (MilliporeSigma), and 0.2 *μ*M estradiol (MilliporeSigma), Serum-free HMEC media (SFM) contained MEM Alpha/Ham's F-12 and 1% Pen/Strep. Immortalized human keratinocytes (HaCaT cells, courtesy of N. Fusenig, DKFZ, Heidelberg, Germany) were maintained at 37 °C, 5% CO_2_ in high-glucose Dulbecco's modified Eagle medium (DMEM) supplemented with 10% FBS and 1% Pen/Strep. HaCaT SFM was DMEM and 1% Pen/Strep. To transduce HMECs and HaCaTs, ERKKTRClover and H2BmRFP lentiviruses were mixed with full-serum HMEC media at a 1:1:2 ratio plus 10 ug/ml polybrene (Santa Cruz Biotechnology, Dallas, TX) and applied to sparsely seed HMECs in a six-well plate. Cells were transduced with the lentivirus mix overnight. After transduction, cells were changed to fresh growth media, passaged twice, and sorted on a BD FACSAria Cell sorter for the Clover and RFP double-positive population. Sorted HMECs were cultured using the same media as the parental line. To confirm the ERKKTR worked as expected, HMECs were seeded on tissue culture plates and treated with 60 *μ*M FR180204 (an ERK inhibitor) for 30 min, 2 ng/ml EGF for 5 min, or 100 ng/ml EGF for 5 min. To seed cells into devices, HMECs were incubated with 2.5% trypsin-EDTA for 10 min at 37 °C and 5% CO_2_, resuspended in full-serum media, centrifuged at 1000 RPM for 5 min, and resuspended at 900 000 cells/ml in full-serum media. To seed cells in devices, HaCaTs were incubated with 0.05% ethylenediaminetetraacetic acid for 15 min at 37 °C followed by 0.05% trypsin for 5 min at 37 °C. Cells were neutralized with 0.5 mg/ml soybean trypsin inhibitor and resuspended at 1500 000 cells/ml in full-serum media.

### Device seeding

Prior to cell culture experiments, full-serum media was flowed through the device and then 2% type B gelatin (MilliporeSigma) was passively flown through the cell seeding inlet^21^ and devices were coated overnight at room temperature. To wash out excess gelatin, full-serum media was flown through the devices and passive pumped through the cell inlet port. Next, full-serum media was placed on the cell inlet and outlet ports and devices were degassed for 30 min, with the vacuum released over a 5-min period to reduce the risk of contamination. Cells were passively pumped through the cell seeding port. Cells were allowed to attach for 24 h, and then serum starved by flowing SFM through the inlet ports and the cell seeding inlet. Cells were serum starved for 24 h followed by initiation of flow in the microfluidic device. Cells were re-fed with either full-serum media or SFM every 12 to 16 h during seeding and serum starvation.

### Device operation

SFM was degassed for 45 min through a Steriflip-GV filter (MilliporeSigma) to prevent contamination. Model 1001 1 ml, 22-gauge blunt tip glass syringes (Hamilton, Reno, NV) were attached to four feet of polytetrafluoroethylene (PTFE) tubing (0.026″ I.D. × 0.050″ O.D, Scientific Commodities, Lake Havasu City, Arizona) and filled with 70% ethanol. The end of the tubing was attached to a vacuum, 5 ml of phosphate buffered saline (PBS) was pulled through the syringe and tubing followed by 4 ml of SFM ± EGF (488 AlexaFluor 10 KDa dextran was added to EGF-containing media or the second stream of SFM at a final concentration of 100 nM). Tubing was attached to device inlet ports, and syringes were placed in NE1000 programmable syringe pumps (New Era, Farmingdale, NY). The coverslip with microdevices was secured in a custom milled OmniTray [ESI, Fig. S1(C)] using double sided foam tape (Grainger, Lake Forest, IL). Devices were placed in a Zeiss Axio Observer.Z1 inverted microscope with an AxioCam 506 mono camera, Plan-Apochromat 20 × 0.8-NA air objective, environmental stage and Zen2 software (Zeiss; Oberkochen, Germany) and filled tubing was connected to the inlet ports [ESI, Fig. S1(D)]. Devices were incubated at 5% CO_2_ and 37 °C. Flow rates of the SFM and growth factor streams were controlled by modulating the rates at which the syringe pump moved the syringe plunger. Based on the syringe inner diameter, the rate at which the plunger is moved is converted to a flow rate. The syringe pumps can be programmed to pause and begin pumping at defined times, administer a steady flow rate, or flow rates that increase or decrease over time. To achieve an increasing concentration, the flow rate of the SFM and growth factor (GF) stream were varied while keeping the overall flow rate constant at 0.36 *μ*l/min. Fluorescent and brightfield images were taken every 1–10 min. Excitation/emission for fluorescently tagged dextran was 365/445, 250 ms exposure at 50% power, ERKKTR was 470/525, 250 ms exposure at 50% power, and H2B RFP was 575/660, 250 ms exposure at 50% power. When using the florescent intensity to confirm a concentration was successfully administered, the signal was normalized to the maximum and minimum signal throughout the time-course. Due to the potential device-to-device variation, the experimental rate of change of the dextran signal over the first hour was quantified and used rather than the programmed rate of change.

### Cell viability

After serum starvation and 28 h of SFM flow (mimicking a full experimental time course), 2 *μ*M calcein AM and 2 *μ*M ethidium homodimer were passively flowed through the cell inlet and cells were incubated for 30 min. SFM was then flowed through the device, and the images were collected on the Zeiss scope as above.

### ERKKTR analysis

Using a custom ImageJ plugin, nuclear images were contrast enhanced and background subtracted. Nuclei were transformed to binary images and used to determine the fluorescent intensity of the ERKKTR signal in a five-pixel ring right outside (C, for cytoplasmic) and inside the nucleus (N, for nuclear). The ImageJ track-mate plugin^22^ was used to identify cell tracks. Using a custom MATLAB© (2016b) code, we extracted ERK activity tracks C/(C + N) ratio into a matrix where rows represented individual cells and columns represented each time point. The C/(C + N) from treatment with FR180204 (∼0.3) and 100 ng/ml EGF (∼0.65) were used to establish the dynamic range of the reporter. Based on this range, cells were classified as active when C/(C + N) was greater than 0.5.^23^

### Immunostaining and imaging in microdevices

At designated timepoints, the experiment was stopped by disconnecting the devices from the syringe pumps, passive pumping PBS through the device, and passive pumping 4% paraformaldehyde (PFA; Electron Microscopy Sciences, Hatfield, PA), followed by a 5 min incubation. The top of the device was then cut off, and 50 *μ*l of 4% PFA was applied to the cells for an additional 25 min and cells were placed on a Belly Dancer shaker at a setting of 2 (IBI Scientific). Cells were then washed four times with PBS. To minimize PDMS interference with immunostaining, the remainder of the device was removed with a razor. The staining protocol used was adapted from a previous protocol,^24^ which allows fluorophores to be eluted so that multiple rounds of staining can be conducted on the same samples. Briefly, cells were permeabilized for 1 h with 0.3% Triton X (MilliporeSigma), 5% goat serum, and 50 mM maleimide in PBS. Primary and secondary antibodies were diluted in 0.3% Triton X, 1% bovine serum albumin (BSA) in PBS. Samples were incubated in primary antibodies overnight at 4 °C on the Belly Dancer shaker, washed four times with PBS, and incubated with secondary antibodies for 2 h at room temperature on a Belly Dancer shaker. For EGFR, the primary antibody was mouse anti-EGFR (Abcam, catalog No. ab30, 1:500), with a secondary antibody of goat anti-mouse AlexaFluor647 (1:500). Nuclei were counterstained with Hoechst 33258 at 1:1000 in PBS for 5 min. For FRA-1, the primary was mouse-anti-FRA-1 (Santa Cruz Biotechnology sc-28310, 1:400), with a secondary of goat anti-mouse AlexaFluor647 (1:200); nuclei were imaged using the H2B-RFP label. Cells were then washed with PBS four times, 10 *μ*l of imaging buffer (700 mM n-acetyl cystine) in PBS was applied to cells, a 9 × 9 mm^2^ coverslip was overlaid and sealed with clear nail polish (VWR), and the cells were imaged with a Zeiss Axio Observer.Z1 inverted microscope with an AxioCam 506 mono camera, EC Plan-neofluar 40×/1.3 oil objective, and Zen 2 software. Excitation/emission for nuclei was 365/445, 50 ms exposure at 10% power, excitation/emission for EGFR was 640/690, 500 ms exposure at 50% power, and excitation/emission for FRA-1 was 640/690, 50 ms exposure at 50% power.

### Quantification of immunostaining

To quantify single-cell intensities of EGFR, images were thresholded in ImageJ^25^ such that controls without primary antibodies had no detectable signal. Cell boundaries were manually drawn. CellProfiler^26^ was used to measure the mean fluorescence intensity of EGFR in individual cells using the cell mask. EGFR intensities were normalized to the median intensities for cells in the SFM condition. Immunostaining results were repeated with identical trends.

### Statistical analysis

Data were analyzed (Prism 8.0; GraphPad Software) using a Wilcoxon matched-pairs signed rank test and the Mann–Whitney U comparisons test as indicated; p < 0.05 was considered statistically significant. To determine the potential switch-like behavior, we utilized the option to fit the Hill equation in Prism.

## SUPPLEMENTARY MATERIAL

See the supplementary material file 1 for supplementary material Figs. S1–S8.

## Data Availability

The data that support the findings of this study and Adobe files for the microfluidic device master are available from the corresponding authors upon reasonable request.
